# Supplementary material for: Reinforcing intensive motherhood: A study of gender bias in parental responsibilities allocation by large language models
Source: PLoS One. 2025 Nov 19;20(11):e0335706. doi: 10.1371/journal.pone.0335706 (PMC12629417; doi:10.1371/journal.pone.0335706)
Supplement: Attachment — Submitted filename: review_intensive_motherhood.pdf [file pone.0335706.s002.pdf]

Review of the manuscript "Reinforcing Intensive Motherhood: A Study of Gender Bias in Parental Responsibilities Allocation by Large Language Models"

Context and contribution

Building on the idea that Large Language Models (LLMs) may reproduce social stereotypes, the article investigates whether two frontier systems (GPT-4.1 and DeepSeek-V3) implicitly endorse the ideology of intensive mothering -- the culturally dominant expectation that mothers shoulder the bulk of childcare. Using a 3-factor experiment that crosses (i) model type, (ii) caregiver role (mother, father, neutral parent) and (iii) responsibility framing (prescriptive vs. descriptive), the authors show that both LLMs assign systematically higher caregiving scores to mothers and that prescriptive prompts elicit stronger attributions of responsibility than descriptive prompts. Mediation analysis suggests these effects are not driven by the models' gender-equality attitudes but rather by associations embedded in training corpora. By extending bias research into the domestic sphere, the study highlights the risk that advanced language technologies may reinforce traditional gender norms in private life and argues for gender-sensitive model design and data curation.

1. Major (general) issues

- Missing page numbers: The PDF has no pagination, which makes it difficult for reviewers to reference specific passages. Please insert page numbers before an eventual resubmission.
- Data availability statement: The link provided (https://osf.io/nzs49/?view_only=7746022768c1455bb2a970b14c7d6fcb) leads to an Unauthorized page.
- Citation formatting: The phrase "(Hays, 1996) concept of intensive mothering" needs correction.

2. Specific comments

#	Section	Comment
2.1	Introduction	Several key terms--intensive mothering, prescriptive advocacy, descriptive advocacy, and the distinction between “ideal” vs. “actual” responsibilities--are mentioned but not defined. PLOS ONE has a broad readership; add concise definitions or footnotes.
2.2	Motivation	The manuscript implies that parents rely on LLM-powered tools, but the mechanism connecting model outputs to real childcare decisions remains implicit. Brief statistics on global AI adoption in parenting (e.g., UNESCO 2024) would ground the argument.
2.3	Hypotheses	H1 references “caregiving responsibility scores” yet the construction of these scores only reappears deep in the Methods section. Move a short description to the hypothesis paragraph.
2.4	Experimental settings	The “temperature” parameter (set to 1.0) is jargon. Add a one-sentence explanation: e.g., “Temperature controls the randomness of generated text--higher values yield more varied but potentially less coherent responses.”
2.5	PROCESS macro	The mediation test is summarized in a single sentence. Readers unfamiliar with Hayes’ PROCESS require more detail: model number, bootstrapping strategy, indirect-effect confidence intervals, and the operationalization of “gender-role beliefs.” A short appendix or table would suffice.
2.6	Table/figure clarity	Results tables should list exact p-values or 95 % confidence intervals rather than qualitative descriptors (“obvious gender bias”). Figures illustrating mean scores with error bars would greatly aid interpretation.

3. Minor editing suggestions

- Replace long em-dashes with the journal’s preferred punctuation or use “--” throughout, for consistency.
- Standardize American vs. British spelling (e.g., “behavior” vs. “behaviour”).
- Check subject-verb agreement in long sentences (e.g., “LLMs were likely driven” → “LLM outputs were likely driven”).

4. Recommendation

The manuscript addresses a timely and socially significant question and employs a creative experimental design. However, the current draft is conceptually under-explained and methodologically terse, which obscures its contribution. With clearer definitions, fuller reporting of the mediation procedure, restoration of the data link, and improved formatting, the study has strong potential for publication in PLOS ONE.

I therefore recommend major revisions before the article can be considered for acceptance.
